# Epidemiological and clinical profile, and survival of patients followed for breast cancer between 2010 and 2015 at the Yaounde General Hospital, Cameroon

**DOI:** 10.11604/pamj.2021.39.182.26866

**Published:** 2021-07-07

**Authors:** Stéphane Zingue, Etienne Okobalemba Atenguena, Laure Leka Zingue, Alain Brice Tueche, Dieudonné Njamen, Alexandre Benjamin Nkoum, Paul Ndom

**Affiliations:** 1 Department of Medical and Biomedical Engineering, Higher Technical Teachers´ Training College, University of Yaounde I, P.O. Box 886 Ebolowa, Cameroon,; 2 Oncology Division, Yaoundé General Hospital, Yaoundé, Cameroon,; 3 School of Health Sciences, Catholic University of Central Africa, Yaoundé, Cameroon,; 4 Department of Internal Medicine, Faculty of Medicine and Biomedical Sciences, University of Yaoundé I, Yaoundé, Cameroon,; 5 Department of Animal Biology and Physiology, Faculty of Science, University of Yaounde I, P.O. Box 812 Yaounde, Cameroon

**Keywords:** Breast cancer, epidemiological profile, clinical profile, overall survival, Yaoundé General Hospital

## Abstract

**Introduction:**

approximately 6000 Cameroonian women died of cancer in 2018, and the breast is the most affected with 2625 new cases. The aim of this study was to establish a pattern of malignant breast tumours in Yaoundé (Cameroon).

**Methods:**

this study was a descriptive and analytical retrospective study of breast cancer between January 2010 and December 2015 in Yaoundé General Hospital (YGH) after the Institutional ethics committee approval. The variables studied were the socio-demographic characteristics, risk factors for breast cancer, types of tumours and type of treatments. The 5-year survival was analyzed by the Kaplan-Meier method. The adjusted hazard ratios and their 95% confidence intervals were calculated to assess the association between studied variables and patient survival through the cox regression using SPSS 23 software. The difference was considered significant at p < 0.05.

**Results:**

among the 344 files collected in this study, breast cancer patients were predominantly female (96.64%, n = 288) aged 45.39 ± 13.35 years, with invasive ductal carcinoma (68.03%, n = 270), located in the left breast (52%, n= 147). The average tumour size was ~6.5 ± 0.3 cm and diagnosed in grade II of Scarf Bloom Richardson (SBR) in 60% (n= 150) of cases. The 5-year survival was 43.3%. Factors associated with this poor survival were the religion (aHR 5.05, 95% CI: 1.57 - 16.25; p = 0.007 for animist and aHR 4.2, 95% CI: 1.53 - 11.46; p = 0.005 for protestant), location of the tumour (aHR 6.24, 95% CI: 1.58 - 24.60; p = 0.012), tumor height (aHR 0.21, 95% CI: 0.04 - 1.11; p = 0.011) and the time spent before medical treatment (aHR 5.12, 95% CI: 0.39 - 8.38; p = 0.011).

**Conclusion:**

the young age, large tumour size and high histological grade in our studied population suggest a weak awareness of women about breast cancer. Action should be taken in early screening to improve the management of breast cancer in Cameroon.

## Introduction

The aging of populations is a real demographic revolution. Since the middle of the eighteenth century, the progress made by humanity in the fields of medicine, housing and nutrition has contributed to the improvement of the quality of life of the populations and, consequently, to the aging of the population [1]. However, the increase of the life span of women is followed by many health problems, called diseases of aging. Some authors have shown that these changes in the lifestyle associated with urbanization and the concomitant loss of traditional protective factors in our African societies are positively correlated with the increased incidence of cancer [2].

Cancer can be defined as a heterogeneous group of neoplastic disorders characterized by an anarchic proliferation of monoclonal cells having acquired a transformed phenotype. It is the second largest cause of death worldwide, accounting for more than 9.6 million deaths per year (13% of all deaths worldwide). Breast cancer is the most frequently diagnosed cancer and the leading cause of death from cancer in women worldwide (626, 679 deaths per year) [2, 3]. The overall survival at 5 and 10 years for the breast cancer in Cameroon is estimated to be 30% and 13.2% respectively and is much lower than that of patients in some developed countries, which is between 90% and 82% respectively at 5 and 10 years [4].

Numerous studies have shown that early diagnosis of cancer allows effective management at a lower cost and improve the overall survival of patients with breast cancer [5, 6]. In Cameroon, due to economic constraints, cultural reasons, the absence of systematic screening policies and also the lack of means of exploration, the management of breast cancer is difficult [7]. This explains why the overall survival at 5 years in Cameroon was found around 30% between January 1992 and December 2010 [4]. In addition, death registration is often incomplete and the recorded cause of death may be inaccurate or missing in this country [8]. Since the scientific data are helpful to take good decisions to ensure improved and equitable cancer care, the present study was undertaken to identify the factors that characterize the overall survival at 5 years in patients with breast cancer at the YGH between January 2010 and December 2015.

## Methods

**Type of study:** this study was a retrospective review of the digital medical records of breast cancer patients followed up at the YGH between January 2010 and December 2015. The study protocols were approved by the Catholic University of Central Africa Institutional Ethical Committee, Cameroon. Patient consent was not required in this retrospective study.

**Presentation of YGH:** the YGH is one of the most specialized hospitals in the treatment of cancer in Cameroon. It has a number of services specialized in the treatment of cancers such as Radiotherapy, Medical Oncology, Anatomic Pathology, Nuclear medicine, Gynecology and Surgery. Breast cancer patients followed up at the YGH were most often referred from other health facilities and came from all parts of the country.

**Sampling:** all patients diagnosed with a breast cancer and registered at the YGH during the study period (2010-2015) were considered. A total of 344 medical records were included in this study after a successive and consecutive recruitment of all patient medical records found in the different services. The medical records excluded were those of patients diagnosed with a benign breast tumour, or those whose medical records were not found or incomplete, as well as patients who were not followed up after the diagnosis of their breast cancer.

**Study design:** all patient medical records obtained in the 5 years study period were reviewed. Collected and analysed data included: the socio-demographic characteristics (age, level of education, reproductive and menopausal status), clinical manifestations (type of tumour, histologic type, histologic grade, type of treatment, severity, lethality, curability). The survival delay of patients within 5 years from the date of unequivocal diagnosis of cancer was obtained by active and passive methods. The passive assessment method of survival was based on medical records. Survival was calculated as the time between the date of diagnosis of cancer and the date of death from any cause or the date of loss to follow up or the date of last follow-up. The active measure consisted to contact the patient or their relatives by phone when the death information were lacking in their medical records. The overall survival at 5 years was calculated by the Kaplan Meier method using SPSS 23 software. Once the data collection was completed, we verified the completeness and likelihood of the information obtained on the collection sheets. We then created an input mask with Epi Info 7 software.

**Data analysis:** once our data matrix was obtained, it was transferred in “Statistical Package for Social Sciences” (SPSS 23) software for statistical analysis. We report descriptive categorical data with percentage and analysis with Fisher´s exact test, descriptive numerical data with mean and standard deviation, and cox regression univariate analysis was performed. P-values less than 0.1 were considered statistically significant to include the retained variable along with their relative modality in the Cox regression multivariate analysis. Survival was calculated by the Kaplan-Meier method. The adjusted hazard ratios were calculated with their 95% confidence interval, in order to assess the influence between the different variables and survival at 5 years.

## Results

**Figure 1:**
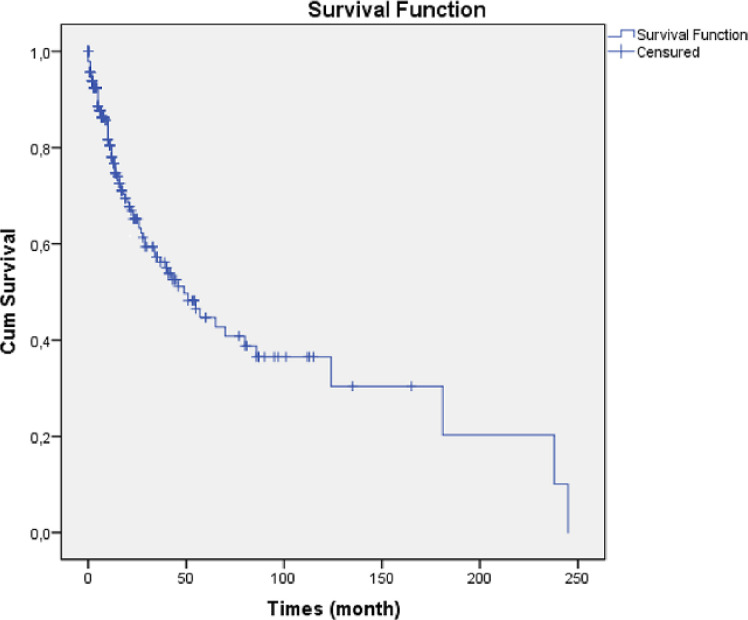
survival curve at 5 years

**Survival at 5 years:** among the 344 files collected in this study, 110 cases (68.2%) had died. The overall survival estimated by the Kaplan Meier method is shown in Figure 1. It can be observed that 285 patients (82.3%) survive more than 1 year, 182 (52.93%) survive more than 3 years and only 149 (43.3%) survive 5 years after the diagnosis of their cancer.

**General and clinical patient characteristics:** the results depicted in Table 1 show that 211 (73%) patients lived in urban areas, while the majority (59 cases, 76%) of patients who succumbed to their cancer before 5 years were in the rural areas. The religions most represented are respectively Catholics (148 cases, 52%) and Protestants (70 cases, 25%). Patients are mostly married (170 cases, 59%). There were 73 (34%) patients in the large “Centre” group and 80 (37%) patients in the “West” group. Ten (3%) patients enrolled in breast cancer during the study period were males. The majority of patients did not have paid employment, we recorded 115 (39%) housewives and 34 (12%) are unemployed. Table 1 also summarizes the bivariate analysis between the general and clinical characteristics and the overall survival at 5 years. Ten (10) variables were analyzed and only the religion was significantly (p = 0.02) associated with the survival at 5 years. The patients belonging to the Islamic and animist religion will be 2.59 times (95% CI: 1.34 - 5.02; p = 0.005) and 2.42 times (95% CI: 1.12 - 5.23; p = 0.024), respectively more likely to die before the 5 years as compared to the catholic.

**Table 1:** bivariate analysis between characteristics of patients and survival at 5 years

Variables	Total	Survivor	Deceased	p-value
**Age (year)**				0.930
< 30	27	11 (41)	16 (59)	
30 to 40	86	36 (42)	50 (58)	
40 to 50	78	35 (45)	43 (55)	
50 to 60	70	32 (46)	38 (54)	
> 60	32	9 (29)	23 (71)	
**Area of residence**				0.866
Urban	211	101 (48)	110 (52)	
Rural	78	19 (24)	59 (76)	
**Religion**				**0.022**
Catholic	148	64 (43)	84 (57)	
Protestant	70	32 (46)	38 (54)	
Islamic	29	10 (35)	19 (65)	
Animist	37	17 (46)	20 (54)	
**Marital status**				0.459
Single	84	35 (42)	49 (58)	
Married	170	75 (44)	95 (56)	
Widow	32	12 (38)	20 (62)	
**Region of origin**				0.312
Centre	73	34 (47)	39 (53)	
West	80	34 (43)	46 (57)	
North	23	10 (44)	13 (56)	
South	34	16 (47)	18 (53)	
Foreign	5	1 (20)	4 (80)	
**Sex**				0.295
Male	10	5 (50)	5 (50)	
Female	288	121 (42)	167 (58)	
**Occupation**				0.394
Civil servant	65	35 (54)	30 (46)	
Private sector	61	22 (36)	39 (64)	
Retirer	13	4 (31)	9 (69)	
Household	115	41 (36)	74 (64)	
Unemployed	39	22 (56)	17 (44)	
**Menarche age (year)**				0.646
< 13	23	10 (44)	13 (56)	
14 to 16	63	25 (40)	38 (60)	
> 17	13	4 (31)	9 (69)	
**Age of 1^st^ pregnancy**				0.180
< 20 years	33	14 (42)	19 (58)	
20 to 24 years	72	23 (32)	49 (68)	
25 to 29 years	20	6 (30)	14 (70)	
≥ 30 years	16	8 (50)	8 (50)	
**Menopause**				0.270
Yes	96	44 (46)	52 (54)	
No	165	65 (39)	100 (61)	

**Risk factor of cancer and histopathological features:** the Table 1 also shows that the average age of patients was 45.39 ± 13.35 years, with a range of 13.58 and 84.39 years. The majority (72; 51%) of patients had their first pregnancy between 20 and 24 years. It was found that 96 (38%) patients were already in menopause. It can also be observed in Table 2 that approximately 153 (59%) patients have between 2 and 5 children, while 57 (22%) have minus 2 children. The majority of patients (124 cases, 85%) practiced exclusive breastfeeding and the majority of patients were breastfed less than 4 years throughout their lifetime while only 5 (3%) had a cumulative duration of breastfeeding greater than 16 years.

**Table 2:** bivariate analysis between histopathological features and survival at 5 years

Variables	Total	Survivor	Deceased	p-value
**Location of the tumor**				**0.075**
Left breast	147	60 (41)	87 (59)	
Right breast	123	52 (42)	71 (58)	
Both	20	11 (55)	9 (45)	
**Tumor height**				**0.021**
< 5 cm	111	50 (45)	61 (55)	
6 to 10 cm	70	25 (36)	45 (64)	
> 10 cm	32	10 (31)	22 (69)	
**Breast pain**				0.164
No	157	65 (41)	92 (59)	
Yes	128	54 (42)	74 (58)	
**Nipple discharge**				0.159
No	259	101 (39)	158 (61)	
Yes	20	12 (60)	8 (40)	
**2nd breast affected**				0.215
No	262	105 (40)	157 (60)	
Yes	35	21 (60)	14 (40)	
**Type of carcinoma**				0.345
Invasive ductal	270	113 (42)	157 (58)	
Invasive lobula	15	7 (46)	8 (53)	
Other	9	4 (44)	5 (55)	
**SBR grade**				**0.066**
Grade I	34	15 (44)	19 (56)	
Grade II	150	69 (46)	81 (54)	
Grade III	66	26 (39)	40 (61)	
**Cancer associated**				0.896
None	291	121 (42)	170 (58)	
Uterine	3	2 (67)	1 (33)	
Ovaries	2	2 (100)	0	
**Associated pathology**				0.467
HIV	10	4 (40)	6 (60)	
HBP	21	9 (42)	12 (57)	
Other	14	7 (50)	7 (50)	
None	137	63 (46)	74 (54)	
**Number of children**				0.172
≤ 1	57	25 (44)	32 (56)	
2 to 5	153	96 (63)	57 (37)	
6 to 10	50	24 (48)	26 (52)	
**Cumul breastfeeding**				0.412
< 50 months	72	28 (39)	44 (61)	
51 to 100 months	47	16 (34)	31 (66)	
101 to 150 months	30	7 (23)	23 (77)	
151 to 200 months	11	5 (45)	6 (54)	
> 200 months	5	2 (40)	3 (60)	

HR = hazard ration, CI = Confidence interval; the % are in the brackets.

The Table 2 also shows that the left breast was the most affected (147 cases, 51%), while only 20 patients (7%) have cancer on both breasts. It was observed that in 35 cases (35%) the contro-lateral breast is affected. Moreover, the average tumour size was big (6.5 ± 0.27 cm) and painful in 128 cases (45%). The majority (270 cases, 79%) of tumors was invasive ductal carcinoma, followed by the lobular carcinoma type (15 cases; 5%). The majority of tumours are detected in SBR Grade II (150 cases, 60%) and SBR Grade III (66, 26%). The majority of patients had no associated cancer (291, 98%) and no pathology (137, 80%). However, the most commonly encountered pathologies were high blood pressure in 21 (13%) patients and HIV in 10 (6%) patients. It further appears from Table 2 that following the bivariate analysis only tree variables (location of the tumour, tumour height and SBR grade) out of the eleven analyzed are significantly associated with the overall survival at 5 years. In fact, patients with big tumour (≥ 10 cm) were 1.34 times (95% CI: 0.30 - 6.02; p = 0.021) more likely to die before 5 years than those with a tumour < 5 cm. Moreover, patients with both affected breasts were 1.4 times (95% CI: 0.75 - 2.75; p = 0.091) more likely to die before 5 years than those with one breast affected. Furthermore, patients with a histological SBR grade III were 2.05 times (95% CI: 0.92 - 4.57; p = 0.078) more likely to die before 5 years than those with the SBR grade I.

**Management of the cancer:**Table 3 depicts the details of the management of patients with breast cancer at YGH from 2010 to 2015. It was found that 234 (79%) patients discovered their tumour by auto palpation, although in 61 (21%) cases the discovery of the tumour was fortuitous. In 200 (68%) cases, the basis of the diagnosis was a biopsy. The majority (105, 26%) of patients have to consult 3 months after the onset of the disease, however, 80 waited more than one year to be consulted. Otherwise, 147 (52%) patients began the treatment 3 months after the diagnosis of their cancer, while 67 (24%) wait one year to begin their treatment. Chemotherapy was the most administered type of treatment observed in 111 (39%) cases followed closely by the combination of chemotherapy with surgery (93 cases, 33%). Table 4 also shows that out of the five studied variables, two were significantly associated with survival at 5 years. They are the circumstance of the tumour's discovery and the delay before treatment. Indeed, patients who discovered their tumours fortuitously have 1.64 times (95% CI: 1.01 - 2.61; p = 0.037) more risk of dying before 5 years. Patients who had waited more than 12 months to start treatment after the cancer was diagnosed had 6.37 times (95% CI: 3.74 - 7.13; p = 0.001) risk of dying before 5 years as compared to patients who started treatment < 1 month.

**Table 3:** bivariate analysis between management of breast cancer and survival at 5 years

Variables	Total	Survivor	Deceased	p-value
**Tumor discovery**				**0.037**
Screening	234	94 (40)	140 (60)	
Fortuitous	61	29 (48)	32 (52)	
**Diagnostic base**				0.807
Biopsy	200	92 (46)	108 (54)	
Needle aspiration	93	32 (35)	61 (65)	
Clinic	3	0	3 (100)	
**Diagnostic delay**				0.213
< 1 month	29	10 (34)	19 (65)	
1 to 3 months	76	33 (43)	43 (57)	
3 to 6 months	47	19 (40)	28 (60)	
6 to 9 months	32	13(41)	19 (59)	
9 to 12 months	25	9 (36)	16 (64)	
> 12 months	80	38 (49)	41 (51)	
**Delay before treatment**				**0.015**
< 1 month	80	15 (19)	65 (81)	
1 to 3 months	67	22 (33)	45 (67)	
3 to 6 months	33	12 (36)	21 (64)	
6 to 9 months	18	5 (28)	13 (72)	
9 to 12 months	12	9 (67)	3 (33)	
> 12 months	55	53 (96)	2 (4)	
**Type of treatment**				0.272
Chemotherapy	111	32 (29)	79 (71)	
Surgery	8	2 (25)	6 (75)	
Hormonotherapy	2	1 (50)	1 (50)	
Chemotherapy + Surgery	93	40 (43)	53 (57)	
Radiotherapy + Chemothe	15	11 (73)	4 (27)	
Chemo + Surgery + Radio	32	14 (44)	18 (56)	
Chemo + Surgery + Horm	25	24 (96)	1 (04)	

HR = hazard ration, CI = Confidence interval; the % are in the brackets

**Table 4:** cox regression multivariate analysis of factors associated with poor survival

Variables	Univariable analysis		Multivariable analysis	
	HR [95% CI]	p-value	aHR [95% CI]	p-value
**Religion**		**0.022**		**0.014**
Catholic	1		1	
Protestant	1.16 [0.69 1.98]	0.570	4.2 [1.53 11.46]	0.005
Islamic	2.59 [1.34 5.02]	0.005	2.68 [0.75 9.52]	0.129
Animist	2.42 [1.12 5.23]	0.024	5.05 [1.57 16.25]	0.007
**Location of the tumor**		**0.075**		**0.012**
Left breast	1		1	
Right breast	0.67 [0.42 1.11]	0.290	0.67 [0.30 1.48]	0.323
Both	1.4 [0.74 2.75]	0.091	6.24 [1.58 24.60]	0.009
**Tumor height**		**0.021**		**0.011**
< 5 cm	1		1	
6 to 10 cm	0.94 [0.22 4.11]	0.930	0.47 [0.09 2.58]	0.385
> 10 cm	1.34 [0.30 6.02]	0.021	0.21 [0.04 1.11]	0.065
**SBR grade**		**0.066**		0.241
Grade I	1		1	
Grade II	1.21 [0.57 2.56]	0.624	2.24 [0.83 6.08]	0.112
Grade III	2.05 [0.92 4.57]	0.078	2.48 [0.75 8.12]	0.135
**Tumor discovery**		**0.037**		0.711
Screening	1		1	
Fortuitous	1.64 [1.01 2.61]	0.037	1.16 [0.53 2.51]	0.711
**Delay before treatment**		**0.015**		**0.011**
< 1 month	1		1	
1 to 3 months	0.50 [0.27 0.92]	0.026	0.59 [0.24 1.47]	0.260
3 to 6 months	0.86 [0.41 1.77]	0.677	0.25 [0.067 0.95]	0.041
6 to 9 months	0.40 [0.09 1.65]	0.204	0.167 [0.02 1.39]	0.098
9 to 12 months	0.00 [0.00 0.01]	0.962	0.00 [0.00 0.01]	0.972
> 12 months	6.37 [3.74 7.13]	0.001	5.12 [0.39 8.38]	0.0001

aHR = adjusted hazard ratio, CI = Confidence interval

**Final model of cox regression multivariate analysis:** six variables were selected in the final model of our study: religion, location of the tumor, tumor height, SBR grade, tumor discovery and delay before treatment. Significant association with the overall survival at 5 years was found with the religion (aHR 5.05, 95% CI: 1.57 - 16.25; p = 0.007 for animist and aHR 4.2, 95% CI: 1.53 - 11.46; p = 0.005 for protestant), location of the tumour (aHR 6.24, 95% CI: 1.58 - 24.60; p = 0.012), tumor height (aHR 0.21, 95% CI: 0.04 - 1.11; p = 0.011) and the time spent before medical treatment (aHR 5.12, 95% CI: 0.39 - 8.38; p = 0.011).

## Discussion

According to estimation of the World Health Organization, Africa will have 21.7 million new cases of cancer and 13 million deaths in 2030 due to the increase in life expectancy everywhere the world if nothing is done [6]. Cameroon, like other developing countries, is faced with insufficient resources to ensure adequate care for cancer patients, whose magnitude is increasing steadily. In addition, cancer is a public health problem that should be considered in the light of the great morbidity it inflicts on the sick.

The results obtained in this work show that 211 (73%) patients live in urban areas, but the greatest number of patients succumbing to their cancer before 5 years are living in rural environment. Women living in urban zone have a high standard of living and therefore have a breast cancer risk twice as high as those living in rural areas, which is consistent with observations of Ndamba Engbang *et al*. [8]. As suggested by these authors, women living in urban areas may be more prone to breast cancer because of the stress that prevails in large metropolises. But, we can also incriminate dietary habits and life style, which are different in urban and rural zones. Although the incidence of breast cancer is greater in urban zone than in rural areas, rural women have a weaker survival at 5 years. The multiple therapeutic itineraries of these patients, who generally refer to traditional healers, could be in cause.

The religions most represented are respectively Catholics and Protestants. Patients were mostly married and belonging to Centre and West groups. These observations are in line with those of Cameroon Demographic and Health Survey [9], and could be explained by the fact that Yaoundé is predominantly populated by the Betis (Centre) and the Bamileke (West), and these ethnic groups are strongly represented in Cameroon. Ten patients were men, affording a sex ratio (M/F) of 0.03. These values are similar to those reported by Ndamba Engbang *et al*. [8], who found a sex ratio (M/F) of 0.02 over the whole population of Cameroon and those of Diallo [10] who found male breast cancers in Bamako in the range of 1-3%. The majority of women patients (178 out of 315) were aged ~45.4 years with a minimum of 13.6 years and a maximum of 84.4 years. This average is higher than that recorded by Essiben *et al*. [11] in Yaoundé, but lower to that of Ndamba Engbang *et al*. [8] who found 46 ± 15.9 years. This confirms the increasing occurrence of breast cancer in Africa and especially in Cameroon, where we found in our study that 94 (30%) patients had breast cancer between 30 and 40 years these 5 years. It was observed that the majority of neoplasms were located on the left breast (51%) cases and that only 7% had both breasts affected. These are in agreement with several reports showing that the left breast most affected that the right one [8, 12].

Just like Ndamba Engbang *et al*. [8] have found 74% of infiltrating ductal carcinoma in Yaoundé, we found 82% infiltrating ductal carcinoma. The majority of cancers were detected in grade II and grade III. This are divergent with the report of Ly *et al*. [13], who found that the SBR grade III was the most common among women in sub-Saharan Africa. In Bangui, grade II was the most prominent with 58%, while grades I and II were in the same proportions (21%) [14], which is in agreement with our observations. At the Gynecological Obstetrics and Pediatric Hospital in Yaoundé, Essiben *et al*. [11] cumulatively regained grades II and III in 89% of their workforce; while Ndamba Engbang *et al*. [8] found values similar to us (65%) for grade II.

Five-year survival was estimated to be 43.3% in our study, which corroborates with WHO's findings that nearly 60% of patients die within the year of diagnosis in developing countries [15]. Patients with both affected breasts were 1.4 times more likely to die before 5 years than those with one breast affected (p = 0.091). This could be explained by the burden of disease imposed by the two tumors. It was noticed that patients who discovered their cancer incidentally have 1.64 folds more risk of dying before 5 years (p = 0.037) than those who have detected their cancer by practicing auto palpation. In addition, patients who started their treatment ≥ 12 months after the diagnosis of cancer had 6.37 (p = 0.001) fold risk of dying in the next 5 years than those who started their treatment earlier. These are consistent with many reports that the high mortality rates due to breast cancer in sub-Saharan Africa, including Cameroon is due to delayed diagnosis [4]. Indeed, many patients are diagnosed with breast cancer at SBR grades that are often very advanced (Grade II and III), where their vital prognosis is already committed.

The tortuous therapeutic itineraries, and especially the lack of financial resources, can be the cause of this delay of diagnosis and treatment. Indeed, as described by Ndongo *et al*. [16] patients with breast cancer most often use traditional healers, alternative therapies such as prayer groups, exorcism and Chinese medicine. However, chemotherapy was the most common type of conventional therapy and the most prescribed chemotherapy line in the HGY oncology department was the Adriblastine-Endoxan combination found in 69 (37%) cases (data not shown). The access to chemotherapy in Cameroon is very difficult and patient support his treatment charges, causing both individual and household poverty by curbing the patient´s economic output due to prolonged treatment and adverse effects of treatment [15]. According to a survey carried out in Yaoundé in January-February 2015, 80% of the antimitotics registered in the National List of Medicines Essential are unavailable or expenses (~ from XAF 226,780 to 500,000). Since the monthly average expenditure of care for a patient suffering of breast cancer was estimated at ~ XAF 74, 769, and the fact that the majority of patients are housewives (39.25%), students (8.53%) or widows (3.07%), it becomes comprehensible that the survival at 5 years in Cameroon is very weak.

The main limitation of this study is the large proportion of loss to follow up, which resulted in numerous unrecovered medical records. This difficulty in archiving medical records is very common in our hospitals and is a major issue for research in developing countries. However, the results presented in this study give an idea of the overall survival at 5-year of breast cancer patients in Cameroon and the main factors that influence it.

## Conclusion

The patients followed at the HGY for breast cancer from 2010 to 2015 have different socio-demographic and cultural characteristics, and the lifestyle of these patients influences their survival, in particular their religion. The invasive ductal carcinoma was found the predominant type of breast cancer and chemotherapy associated or not to surgery was the most prescribed treatment. The delays in the treatment after diagnosis remain the factor which greatly influences the overall survival at 5 years in Cameroon. Unfortunately, the time between the diagnostic and the start of treatment was found very long because of financial constraints. The young age, large tumour size and high histological grade in our studied population suggest a weak awareness of women about breast cancer. Action should be taken in early screening to improve the management of breast cancer Cameroon.

### What is known about this topic

- It is well known that early diagnosis of the breast cancer allows effective management at a lower cost and improve the overall survival of patients;
- In Cameroon, due to economic constraints, the management of breast cancer is difficult;
- Cameroonian authors reported that the overall survival at 5 years in Cameroon was around 30% between January 1992 and December 2010 [4].

### What this study adds

- This study brings fresh data on the pattern of breast cancer patient in Cameroon and revealed that: they aged 45.39 ± 13.35 years, with invasive ductal carcinoma (68%), located in the left breast (52%);
- The average tumour size was found ~6.5 ± 0.3 cm and diagnosed in grade II of Scarff Bloom Richardson-SBR in 60% of cases;
- The survival at 5 years in Cameroon from 2010 to 2015 was 43.3%, testifying that efforts are undertaken to fight cancer in Cameroon.
